# Incontinence of Urine1Lecture at the Philadelphia Polyclinic.

**Published:** 1893-11

**Authors:** Herbert B. Carpenter

**Affiliations:** Instructor in Clinical Medicine in the Philadelphia Polyclinic


### INCONTINENCE OF URINE.[1]

BY HERBERT B. CARPENTER, M. D.,

Instructor in Clinical Medicine in the Philadelphia Polyclinic.

GENTLEMEN:—This young woman of 18, comes to us complaining, as she describes it, of a weakness of the bladder, which has existed for years. She has involuntary emissions of urine at night during sleep. She has fairly good control through the day, but often has to exert herself. She has menstruated irregularly for three years. She looks poorly nourished and a little anemic. Her tongue is slightly coated, appetite fair and bowels regular.

The examination of the urine is negative.

Now we may recognize several varieties of incontinence of urine. 1st. The incontinence may occur only at night (enuresis nocturna). 2d. It may occur during the day (enuresis diurna). 3d. The bladder may fail to hold the urine both in the day and and night (enuresis continua). Again there are cases in which it occurs only upon some nervous part.

Most cases are seen between the third and the thirteenth year, and a little more frequently in males. The sphincter of the bladder becomes developed towards the end of the second year, but the infantile condition may persist for some time and debilitating diseases occuring in later years may restore it to its original incompetency.

*The causes* are numerous, and the diagnosis of the exact condition of the affairs may be quite difficult.

1st. Cases resulting from debility of the neck of the bladder and the internal sphincter—which is often a part of a gen-

1 Lecture at the Philadelphia Polyclinic.

eral muscular debility, or in other cases the general muscular system is well developed, while the neck of the bladder alone seems to be neglected.

In these cases as Jacobi explains, "The posterior part of the urethra when narcotised as it were, during sleep gives away before the gentliest pressure on the part of the expelling muscle of the bladder." Profound sleep is not a cause of incontinence.

2d. Cases depending on insufficient innervation, diseases of the spinal cord and of the nerve centers, and cases where the reflex apparatus is defective, the sphincter which contracts normally while the bladder is filling up, loses its control.

3d. Reflex incontinence arises from a variety of causes, namely, a contracted prepuce, collections of smegma behind the corona of the glans penis, and rectal irritation, the result of worms, retention of feces. Fissure or polypus is a frequent reflex cause. Cases may depend on an increased reflex irritability of the bladder itself.

4th. Masturbation is also a frequent cause of incontinence of urine.

5th. The over expansion of the bladder with urine is a cause of nocturnal incontinence. Hence the drinking of large quantities of liquids in the evening is to be prohibited.

7th. Incontinence is often due to change in the constitution of the urine. The secretion becoming intensely acid, or loaded with urates and phosphates (the result of improper digestion) irritates the mucous membrane of the bladder, causing contraction of the detrusor muscle and frequent expulsion of urine. Cystitis and stone cause incontinence in the same manner.

In adults we have as additional causes, hypertrophy and diseases of the prostrate gland, fistula after parturition or after operation for stone, the too frequent use of large bougies passed into the bladder. The mechanical distension of the bladder from obstruction, due either to enlarged prostate, stricture of the urethra, etc., so weakens the walls of that organ, as to induce a dribbling or overflow from paresis of the sphincter vesicæ.

*Treatment.*—The causes of enuresis are so numerous that it requires tact and discrimination in the selection of remedies, and a careful examination must be made to discover if there exists any local cause likely to produce and perpetuate the affection. Some cases get well spontaneously about the period of puberty when the whole genito-urinary apparatus undergoes rapid development.

General anemia and muscular debility require a full nutritious diet, massage and electricity (one pole applied to the perineum and the other to the lumbar region) and the use of tonics, especially strychnine and iron in large doses. Cod-liver oil will also be useful. Whenever there is an excess of urates, phosphates, or oxalic acid, or sugar in the urine the source of the disturbance must be attended to.

The diet must be corrected and very often the most obstinate cases will yield when the urine is made clear and mild by the use of alkalies, as sodium bicarbonate or potassium acetate.

To lower the sensibility of the bladder and the cerebro-spinal centers, no remedy is so efficient as belladonna or its alkaloid. Children tolerate very large doses but to be efficient it must be pushed until its physiological effect is produced. To a child of three years you may begin with three drop doses of the tinture.

A single dose of atropia at night, gr. 1-100 to 1-25 will act well. Often a combination of potassium bromide (grs. xxx) and belladonna will be more efficacious than either drug alone. A pill of

Camphor gr. $\frac{1}{2}$–2

Belladonnæ $\frac{1}{12}$–$\frac{1}{4}$

Four times a day to a child of 10 years is useful.

Ext. humuli fl. (♏ v–xv) may be tried.

I have used antipyrin with success in several cases after failing with other drugs in this class.

Reflex causes are to be removed. If there is a contracted prepuce, circumcision, or incision will be required, although at times a gentle dilatation will answer. Firm adhesions of the prepuce will require careful detaching. Intestinal worms must be removed. Constipation is to be relieved by laxatives. Vaginal catarrh, which is often obstinate, is to be treated with dusting powders of borax, tannic acid or astringent injections, and with tonics. Polypoid excrescences in the urethera or vagina are to be removed. Fissure of the rectum to be treated by cauterization, dilatation or incision. The presence of stone in the bladder, fistula, tumor, etc., have their own indications and require surgical aid.

Tincture of cantharides in drop doses three times a day is said to be useful in the incontinence of adult females, on laughing or sudden movement.

In incontinence dependent upon debility of the bladder, turpentine (5–15 drops) or Ext. rhus aromatica fl. (3–15 drops) are sometimes of service. Cases in adults if combined with loss of sexual power have been treated with success by suspension.

In very obstinate cases of urinary incontinence, after other remedies have failed to afford relief, and in cases where there is marked irritability of the neck of the bladder and the prostatic part of the urethera, cauterization by means of a two per cent. solution of nitrate of silver may be tried. An elastic or a metal sound may be introduced into the bladder every third day for two or three minutes, this will be found beneficial alike in the male and female. Sanger has treated a number of cases of incontinence, which he believed to be due to a paresis of the sphincter in women and girls by gentle dilatation, and the manipulations he believed strengthened it.

Some chronic cases, especially those associated with vesical catarrh, may be helped by forcibly dilating the bladder with increasing amounts of water.

Masturbation, which often causes an irritation of the prostatic urethera, will be found difficult to cure. The child will have to be watched and corrected.

Incurable cases in males, should wear a urinal.

The bladder and rectum should both be evacuated before going to bed. If the patient is capable of being reasoned with, he should be advised to sleep upon his side and to rise once or twice during the night and empty the bladder.

In young children, the nurse or parent should be instructed to take the little patient up and rouse it sufficiently to urinate. A spool or large button strapped to the back to prevent the child sleeping on its back is sometimes used by the mother.

The use of collodion over the meatus at night I would not advise, and I have seen serious ulceration from a rubber band and a piece of string tied around the penis to prevent the boy wetting the bed.

To attempt to cure incontinence by intimidation and punishment is brutal, but not uncommon, and nothing is more unfortunate as it only makes matters worse, the fault being beyond the child's control.

To return to our patient I believe her enuresis is due not only to debility of the muscles of the neck of the bladder and sphincter, but also to weakness of the spinal centers governing the bladder. We will give her a tonic of iron and arsenic. And give her a solution of strychnine (1 gtt.=$\frac{1}{120}$ gr.) as it will be much easier to increase the dose in this form. We will begin with gr. $\frac{1}{60}$ and increase to gr. $\frac{1}{8}$ three times a day.

[Six weeks later the girl reported that for over a week she had been able to hold her urine both day and night. She is taking $\frac{1}{10}$ gr. strychnine three times a day and will continue at that dose for a few weeks longer.]—*The Phila. Polyclinic.*

